# LeiP#netz 2.0: mapping COVID-19-related changes in mental health services in the German city of Leipzig

**DOI:** 10.1007/s00127-022-02274-2

**Published:** 2022-03-25

**Authors:** Gesa Solveig Duden, Stefanie Gersdorf, Kai Trautmann, Ingmar Steinhart, Steffi Riedel-Heller, Katarina Stengler

**Affiliations:** 1 Leipzig Heart Institute GmbH, Leipzig, Germany; 2 Community Psychology, University of Hagen (Fernuniversitaet), Hagen, Germany; 3 Institute for Social Psychiatry, Ernst-Moritz-Arndt-University Greifswald, Greifswald, Germany; 4 Institute of Social Medicine, Occupational Health and Public Health, University of Leipzig, Leipzig, Germany; 5 Clinic for Psychiatry, Psychosomatics and Psychotherapy and Centre for Mental Health, Helios-Park-Hospital Leipzig, Leipzig, Germany

**Keywords:** Community psychiatry, COVID-19, Coronavirus, Pandemic, Mental health services, Telepsychiatry

## Abstract

**Purpose:**

The purpose of the study was to investigate the changes in psychosocial and psychiatric services in the German city of Leipzig during the COVID-19-pandemic.

**Methods:**

A participatory, mixed-methods study was used involving a quantitative online survey and qualitative semi-structured interviews with professionals. Quantitative findings were reported with descriptive statistics, and thematic analysis was conducted for qualitative data.

**Results:**

Fifty professionals from various mental health services participated in the survey and eleven professionals were interviewed. Quantitative findings showed that some services were closed intermittently and that there was a stiff increase in use of digital/telephonic service and a decrease in face-to-face services. Staff or funding did not change considerably during the pandemic. Psychosocial groups were suspended or reduced, while access to services became more difficult and professional training for staff was stopped. Thematic analysis of the interviews showed that professionals experienced different phases and levels of change during the pandemic, including changes on a structural level, on the users’ level, and on the staff’ level. Professionals particularly criticised the equivocality of COVID-19 regulations, a defective flow of information and lack of attention for mental healthcare in public policies. They also saw positive aspects, such as the capacity of users and the outpatient care system to adapt to the new situation.

**Conclusion:**

This study suggests directions for policy and service development, such as communicating clearly in infection-control measures, fostering outpatient care and networks between services.

## Introduction

In March 2020, the COVID-19-pandemic started affecting people and institutions worldwide. Governments implemented measures to control the spread of the SARS-CoV-2 virus, such as closure of schools and cultural institutions, physical distancing, quarantine of identified cases, and curfews. Mental health services (MHS) were also impacted [1–4]. As staff and patients needed to self-isolate, some services had to close down completely or reduce their offers, others re-structured their services [4, 5]. At the same time, some MHS found innovative and flexible solutions to guarantee the continuous provision of MHS for people with severe mental illness, such as implementing telepsychiatric services [4, 6].

In Germany, the care situation for patients with psychiatric disorders deteriorated: the inpatient treatment capacity of psychiatric clinics decreased by about 40% compared to the time before the pandemic [7, 8], and emergency hospital admissions and length of hospital stays declined significantly during the first phase of the pandemic (March 13–May 21, 2020; [9]). Day-care and outpatient services were only available in a limited form or were completely put on hold [7]. The pandemic situation led many professionals to expect a surge in mental health problems [10], yet, people with mental illnesses and their interests were rarely mentioned in the German COVID-19 restriction policies [11].

Meanwhile, German mental healthcare (MHC) was facing various challenges before the pandemic already: the provision and funding of MHC in Germany is fragmented [12] with in- and outpatient services being separated on both the organisational and financial level [13]. German psychiatric care of people with severe mental illness still relies to a great extent on inpatient psychiatric hospitals [12, 14]. Community psychiatric models exist as an alternative, but lack support and funding [15] and are, thus, only marginally represented in the German treatment context [16]. For instance, the German health insurance system impedes the nationwide implementation of psychiatric home treatment teams into the MHC system [17]. Meanwhile, inpatient psychiatric care is facing a range of challenges—numbers of patients and readmissions increase while retention periods decrease [18, 19].

Recently, there have been stronger attempts to integrate strategies modifying MHC structures towards a community and outpatient focussed system. An example of such a strategy is the Functional Basic Model for the Psychiatric Care of Persons with Severe Mental Illness (FBM [20, 21]). This model describes minimum standards for the community psychiatric care for people with serious mental illness [22]. The foundations of the FBM lie in the UN Convention on the Rights of Persons with Disabilities, the guideline “Psychosocial therapies in the case of severe mental illnesses” [23] and the evaluation of alternative hospital treatment models from Germany [24]. The FBM is designed to span across various treatment sectors and across the sections of the Code of Social Law. It describes the functions needed for adequate psychiatric care, independently of their institutional forms and funding [20]. The most recent version of the FBM encompasses 16 functions (see Table 1).

**Table 1:** Characteristics and functions of participating services and institutions

Form of service or institution	Survey (*n* = 50)	Interviews (*n* = 11)	Total (*n* = 61)
	*n* (%)	*n* (%)	Total *n* (%)
Psychological counselling centre	13 (26)	4 (36)	17 (28)
Assisted living residence	11 (22)	2 (18)	13 (21)
Psychiatric walk-in-clinic	9 (18)	1 (9)	10 (16)
Social centre [Begegnungstätte]	5 (10)	5 (45)	10 (16)
Rehabilitation institute	8 (16)	1 (9)	9 (15)
Psychiatric day ward	7 (14)	1 (9)	8 (13)
Soziotherapeutic residence	7 (14)	1 (9)	8 (13)
Community social psychiatric centre	5 (10)	3 (27)	8 (13)
Social psychiatric service	6 (12)	1 (9)	7 (12)
Self-help group	4 (8)	3 (27)	7 (12)
Psychiatric hospital	3 (6)	1 (9)	4 (7)
Institution for an additional income	2 (4)	2 (18)	4 (7)
Day structuring services	2 (4)	2 (18)	4 (7)
Workshop for people with disabilities	2 (4)	1 (9)	3 (5)
Neighbourhood centre	1 (2)	2 (18)	3 (5)
Socio-therapy	2 (4)	1 (9)	3 (5)
Consumer survivor initiative	1 (2)	2 (18)	3 (5)
Emergency shelter	–	2 (18)	2 (3)
Medical private practise	1 (2)	–	1 (2)
Vocational training unit	–	1 (9)	1 (2)
Function according to the FBM	n (%)	n (%)	Total n (%)
Counselling	38 (76)	10 (91)	48 (79)
Work with relatives	25 (50)	6 (55)	31 (51)
Health promotion	22 (44)	7 (64)	28 (46)
Multiprofessional treatment	16 (32)	7 (64)	23 (38)
Work with social space	17 (44)	5 (46)	22 (36)
Outpatient (walk-in) treatment	15 (30)	7 (64)	22 (36)
Participation (employment)	12 (24)	9 (82)	22 (36)
Assistance for daytime activities	16 (32)	4 (36)	20 (33)
Mobile and home-visit treatment	13 (26)	7 (64)	20 (33)
Preventative work	16 (32)	3 (27)	19 (31)
Peer work	11 (22)	5 (46)	16 (26)
Participation (education)	6 (12)	8 (73)	14 (23)
Crisis intervention 24/7	7 (14)	7 (64)	14 (23)
Inpatient treatment	6 (12)	7 (64)	13 (21)
Regional coordination	7 (14)	2 (18)	9 (15)
Psychotherapy	6 (12)	2 (18)	8 (13)
Spaces for retreat	6 (12)	1 (9)	7 (12)
Medical rehabilitation	5 (10)	1 (9)	6 (10)
Intensive treatment 24/7	2 (4)	–	2 (3)

A pilot study, LeiP#netz 1.0, set out to map and evaluate these MHS functions in Leipzig—Germany’s eighth biggest city with 597,493 inhabitants [25]. For this purpose, a questionnaire was developed (“Gempsy” [26]) and successfully employed to obtain detailed information on the available MHS in the city and on their functions according to the FBM. The main results showed that particularly crisis and emergency MHC provision, as well as the intersections of in- and outpatient care were insufficient. Meanwhile, the study also found that there was an active network of MHS in the city with established round tables (i.e. stakeholder discussions concerning the work with people with mental illness) and cooperations [27]. These results represent the starting point for the follow-up project LeiP#netz 2.0 which is presented here.

The goal of the present study (LeiP#netz 2.0) was to investigate how the MHS in the German city of Leipzig were affected by the COVID-19-pandemic and its restrictive measures on a structural, staff and content level. Since research which directly assesses the experiences of professionals working in MHC during the COVID-19-pandemic is still underdeveloped [4], the study aimed to look at professionals’ perspectives to identify positive and negative changes caused by the pandemic. This research might help to better meet the needs of psychiatric patients and MHC providers in future crisis situations.

### Research question

Which changes did the MHS and FBM-functions experience due to the COVID-19-pandemic and associated restrictive measures?

## Methods

### Instruments

#### Development

A quantitative questionnaire was developed based on the instrument used in the pilot project, LeiP#netz 1.0 [28]. A qualitative interview guide was created following the guideline by Helfferich [29]. Feedback on the first drafts of instruments was obtained from four project partners, including the coordinator of psychiatric services in Leipzig, Thomas Seyde. The latter is the regional contact person for all areas of psychiatric care and the regional manager responsible for planning, setting up and coordinating the necessary assistance for people with mental illnesses in close cooperation with stakeholders, service providers, and people with mental illness based on the principles of person-centred and goal-oriented service provision. The questionnaire was adapted and converted into an online format using LimeSurvey. Instruments were pilot tested with six professionals (inpatient-clinic psychiatrists, a social worker, a psychologist, the head of a social-psychiatry institute, and the head of a psychiatric clinic), who provided feedback on the understandability, relevance, and uttered their concerns with specific items. Following this, the research team adapted the questions and agreed upon a final version of the instruments.

#### Content

The questionnaire involved 12 subsections relating to (1) data about the participant and the service/institution, (2) changes in services offered, (3) changes in funding, (4) changes in spaces, (5) changes in staff structure, (6) changes in team climate, (7) changes in vocational training, (8) changes in users, (9) changes in content of services, (10) changes in cooperations and networking, (11) data about positive developments evoked by the pandemic and (12) about causal factors of change during the pandemic. Participants were asked between 41 and 60 questions depending on their previous choices and eligibility for branching specific sub-questions. Typically, the survey took 30–45 min to complete. A copy of the survey in German language is available here: https://umfrage.leipnetz2.de/index.php/981543?lang=de.

The interview guide consisted of four questions: “How did your services change during the pandemic?”, “What would you need to be able to work well in future emergency/pandemic situations?”, “What is needed for users to be adequately provided for in such a situation?”, and “What did you learn from your work during the pandemic?”.

### Participants

Participants were professionals who worked in MHC institutions in the city of Leipzig (see Table 1). For the qualitative part, 11 professionals were interviewed. For the quantitative part, 81 people started the survey (including those who clicked ‘Start’ but provided no or minimal data) and 45 got to the end. We report results for participants who completed at least 3 of the 16 pages of the online survey. This produced a sample of 50, which in its totality represented all of the 16 functions of the FBM.

### Ethics

Ethical approval was obtained from the Ethics Committee at the Medical Faculty of the University of Leipzig (Reference number: 044/21-ek) and the Ethics Committee at the Saxon State Medical Association (Reference number: EK-BR-9/21-1). Participants signed an informed consent sheet and data were anonymised and stored securely in accordance with data protection laws.

### Procedure

Participants were recruited by contacting all MHS emanating from the pilot project LeiP#netz 1.0 and additional services identified in public registers resulting in a total number of 77 institutions. Data were collected from March 16 to May 21 2021. During this time, the online survey was administered to all participants and 11 participants additionally took part in semi-structured interviews via Skype, Zoom or MS Teams. Interviews were transcribed verbatim in German language with all identifiers removed. Back-translation to English was used for the interview verbatim extracts presented here. After the first round of data analysis, a network meeting was held in July 2021 with the purpose of providing an additional feedback loop and actively involving participants in the research process. Following a participatory action research approach [30], the meeting offered a space for discussion of preliminary results and for connecting participants among each other.

### Analysis

*Quantitative Data* Descriptive statistics were produced using JASP [31] to summarise relevant aspects of the quantitative data.

*Qualitative Data* We used thematic analysis [32] and the program MAXQDA [33] to analyse the interview transcripts. Two independent coders identified codes (most basic units of meaning in the transcripts) and grouped them into a hierarchical structure (coding tree) consisting of subthemes and themes. Subsequently, the coders compared their codings and coding trees. Levels of agreement between the two coders for randomly selected transcripts were 90%. Disagreements were resolved through consensus discussion. The findings were adjusted in accordance to the additional feedback given by participants at the network meeting.

## Results

Across the qualitative and quantitative studies, 13 (21%) participants indicated having a therapeutic role, 38 (62%) a leadership role and 14 (23%) an administrative role in their institution (multiple responses possible).

**Fig. 1:**
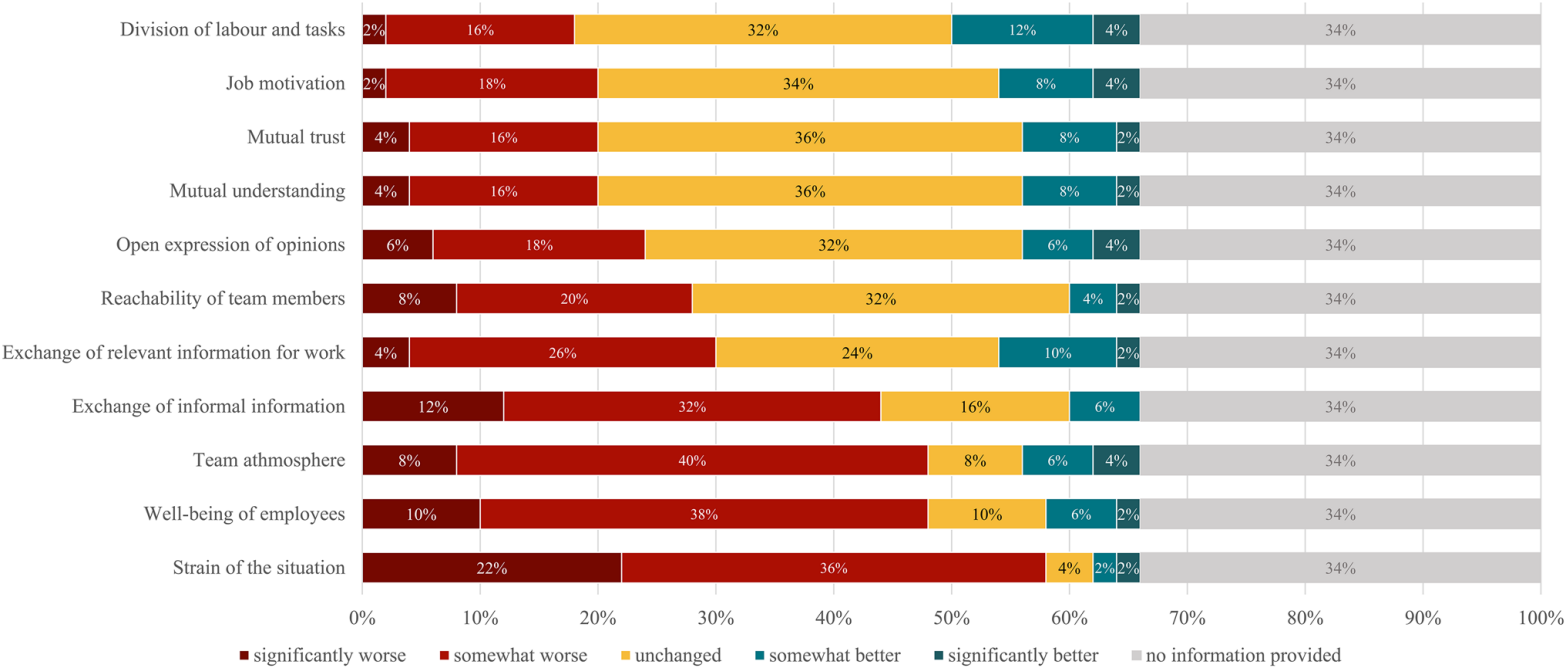
.

The quantitative findings are represented in Tables 2 and 3, and Fig. 1.

**Table 2:** Descriptive statistics (“How did the following aspects change due to the pandemic?”)

	*n* (no response)	*n* completely suspended	Mean (SD)	Median
Phone services	47 (3)	0	4.5 (0.66)	5
Digital services	37 (13)	0	4.32 (0.71)	5
Proportion of users with severe mental illness	50 (0)	–	3.32 (0.65)	3
Individual settings	47 (3)	0	2.98 (0.9)	3
Overall utilisation	50 (0)	–	2.86 (1.09)	3
Inclusion of former psychiatric patients	30 (20)	3	2.7 (0.67)	3
Inclusion of relatives	34 (16)	2	2.59 (0.91)	3
Users (daily)	50 (0)	–	2.12 (1.34)	2
Face-to-face services	49 (1)	2	1.85 (0.75)	2
Group settings	45 (5)	15	1.67 (0.92)	1
Professional training	46 (4)	5	1.46 (0.81)	1

1 = significant decreased; 2 = somewhat decreased; 3 = unchanged; 4 = somewhat increased; 5 = significantly increased

**Table 3:** Descriptive statistics (“Did the following aspects change due to the pandemic?”)

	*n* (no response)	Yes (*n*/%)	No (*n*/%)
Teamwork	47 (3)	33 (70)	14 (30)
Teamclimate	47 (3)	33 (70)	14 (30)
Cooperation with other services	46 (4)	25 (54)	21 (46)
Access to services by users	46 (4)	21 (46)	25 (54)
Users’ concerns	46 (4)	14 (30)	32 (70)
Funding of services	49 (1)	12 (24)	37 (76)
Content and functions of services offered	46 (4)	10 (22)	36 (78)
Crisis management	37 (13)	7 (19)	30 (81)
Composition of employed staff	48 (2)	0	48 (100)
Location of services	49 (1)	0	49 (100)

Percentages are rounded and reported relative to the number of valid responses for each question

The qualitative analysis resulted in eight themes with varying numbers of subthemes (see Table 4).

**Table 4:** Themes and subthemes of the qualitative analysis

Theme	Subtheme
Changes on a structural level	Digitalisation and telemental health
	Discontinuation of MHS vs. open and functioning services
	Worsening of access
	Increase in administration efforts and lack of financial support
	Impairment of professional training
Changes on the users’ level	Negative impacts on well-being
	COVID-19-pandemic as a new topic in therapy
Changes on the staff level	Changes in team climate and teamwork dynamics
	Changes in work procedures and administration
	Decrease of employees’ well-being
Network of MHS	Importance of having a network
	Obstacles to a functioning network
Problematic issues and criticism	Equivocality of Covid-19 regulations
	Defective flow of information
	Lack of attention for MHS
	Deficiencies in digital infrastructure
	Intensification of existing problems
Positive aspects of the pandemic	Increasing awareness for uncontrollable life events
	Flexibility and capacity to adapt
	Learning effects
	Users’ capacity to deal with crisis situations
Wishes for future emergency situations	Overview of available MHS
	Consideration of users’ and relatives’ perspectives
	Specific measures for vulnerable group
	Consistent and clear communication
	Mental health as part of policies
	Financial support of MHS
	Low-threshold access to MHS
	Reflection on how to keep services in emergency situations running
	Outpatient care as the way forward
Four different phases of change	1st lockdown: uncertainty
	Summer 2020: “Normality”
	2nd lockdown: “COVID-19 Fatigue”
	Begin of vaccinations

*MHS* mental health services

### Changes on the structural level

The most reported changes on the structural level concerned digitalisation and telemental health. In the quantitative analysis, 68% indicated an increase in digital services, and 86% in telephonic services caused by the pandemic.

In the qualitative part, on the one hand, positive consequences of the digitalisation were described. These included better and quicker access to services for service users, benefits for people with social anxieties, and professionals overcoming a reluctance to use technologies: “I could never have imagined doing online therapy. And now I think it's a really great medium.” [210504]. On the other hand, professionals voiced concerns regarding digitalisation, such as users’ difficulties to employ digital tools, and exhaustion of employees due to increasingly blurry boundaries between work and home. In addition, some professionals felt that digital work would never be able to replace face-to-face services.

Results demonstrated a discontinuation of MHS due to the pandemic with 78% of survey participants indicating a decrease in face-to-face services, of which 4% reported a complete hold of these services. 30% of services indicated the complete suspension of group activities for MHC users, and 50% that group offers were reduced. Meanwhile, quantitative results also showed that on average, the overall services utilisation, the proportion of users with severe mental illness, as well as the provision of individual settings did not change (Table 2).

Qualitative results explicated that the disruption of MHS particularly concerned psychosocial groups, work involving relatives of users and volunteers, and services focussed on workplace integration and leisure time activity. Meanwhile, participants also indicated MHC continuity with inpatient services, home visits, and social psychiatric services remaining open and functioning.

A change in access to MHS was reported by 46% of survey participants (Table 3). 34% described that now pre-registration was necessary to access services, and 26% indicated an increase in waitlist times. At the same time, 38% reported that newly introduced digital services allowed a new way to access services.

Qualitative results confirmed the findings regarding newly introduced pre-registration of users and increase in waitlist times due to the restrictive measures and remote work of employees. Ad hoc consultation was not possible anymore.

The pandemic resulted in financial issues and augmentation in administration efforts in MHC. For the majority of services (76%, Table 3), there were no changes in funding. Meanwhile, for 32%, it was unclear who would cover the costs of the safety equipment needed due to the pandemic.

Similarly, interview participants reported that the budget of services did not foresee the extra costs of hygiene measures (masks, tests) and some experienced a decrease in financial donations.

The quantitative results also showed that professional training was considerably impaired by the pandemic. For 10%, professional training was completely suspended and 74% reported its (considerable) reduction (Table 2). At the same time, professionals wished for more specialised training in a crisis situation, particularly regarding topics such as self-care and coping with strain for employees (64%), providing support for users afflicted by the crisis (60%) and digital service provision (48%).

Qualitative data confirmed these findings and indicated that if professional training took place, it happened almost exclusively within the organisation and only due to the initiative of engaged employees.

### Changes on the level of users

In the survey, 42% of participants reported a decline in daily service users, 2% an increase, and 32% indicated that numbers remained unchanged. 30% saw particularly people with chronic illnesses, 26% single parents and 24% users older than 51 years as strongly impaired by the COVID-19-pandemic in using MHS. 30% reported that users’ concerns had changed due to the pandemic (see Table 3).

Qualitative data confirmed that due to the pandemic concerns changed. Particularly coping with the COVID-19-pandemic itself became a new topic in therapeutic spaces, and partially hindered the therapeutic work on any other topic: “It was really about the here and now, […] and how to deal with it. But therapy was out of the question.” [210315]. While interview participants mostly reported a decline in users’ numbers, they expected a surge in mental health problems caused by the pandemic in the future. Especially the reduction in social contacts and the prohibition of visitors in inpatient care were seen to negatively affect users’ well-being.

### Changes on the staff level

The survey revealed changes in team climate and teamwork dynamics. 70% reported a transformation in teamwork and team climate, with 62% indicating that face-to-face team meetings had stopped completely or were reduced, 22% stating that digital meetings were newly introduced, and 42% noting that individual work had increased. Figure 1 shows which aspects of the team climate were affected. In particular, 48% reported a negative impact of the pandemic on employees’ well-being and the team atmosphere, and 58% an increase in the strain of work.

Interview participants also reported that the team atmosphere worsened and exchange of informal information declined. Meanwhile, some described an “atmosphere of pioneers” [210503]—a feeling that they had to stick together as a team to meet the new challenges. For instance, professionals embraced communicating with politics as new tasks of their job, as they became aware of the direct effects policies had on their services and users. In the qualitative data too, participants reported on high levels of strain among employees, particularly due to quickly changing rules and processes: “all kinds of information, today the decision is like this, the day after tomorrow like that, which regulation applies to me now, depending on where I live and work, what do I have to pay attention to, now something is different again—so the nerves are on edge.” [210318]. Furthermore, for some professionals having to take care of and home-school their own children constituted additional strain.

### Network of MHS

The quantitative analysis showed that for 54% cooperations with the local MHS network changed due to the pandemic (Table 3). Of these, 77% reported having less contact with other members of the network, and 96% indicated that personal meetings involving professionals from other services were reduced or cancelled. Pandemic-related restrictions and the closure of some services were stated as the main reasons for decreasing network activities. 64% of survey participants wished for improving cooperations in the future.

Interview participants reported that the COVID-19-pandemic became an obstacle to a functioning network of services. Cooperations and communication with each other was placed on hold, network meetings cancelled, other demands increased, and spontaneous and personal encounters stalled. However, the importance of having a network of the MHS in the city was highlighted throughout the interviews.

### Problematic issues and criticism

As problematic issues, in the survey, the deficiencies in available digital equipment for users (70%) and services (58%), and a lack of digital competencies among staff (64%) became apparent, as well was missing attention for MHS in the COVID-19 regulations (54%).

Similarly, interview participants also remarked on the deficiencies in digital infrastructure and on the lack of attention for MHS during the pandemic. As services were not mentioned explicitly in the COVID-regulations, managers were responsible for deciding if services remained open. Interview participants felt that the “stay at home policy” did not work well for people with mental health problems. Furthermore, they uttered criticism especially concerning the equivocality of COVID-19 regulations, the permanent changes of these regulations and the consequential high level of uncertainty among professionals: “…basic requirements were missing. What was allowed, what was not allowed? For example, could we have done a group with five people or could we not have done it if we had had a room that was big enough?” [210319]. The uncertainty resulted in chaotic and ever-changing work procedures, unclear responsibilities, stress among employees and leaders. Professionals criticised that flow of information within and among institutions was defective and that particularly the communication with authorities was impaired. Participants reported that the already existing problems intensified during COVID-19, such as a general lack of specialised professionals.

### Positive aspects of the pandemic

While the negative consequences of the pandemic prevailed in the data, professionals also indicated positive implications. In the questionnaire, 76% reported that the digital competencies of professionals were strengthened, 58% saw a bettering in crisis management of the institution, and 46% an extension of telehealth services as positive consequences of the pandemic.

In the interviews, professionals perceived a high flexibility and capacity to adapt to the new situation particularly in outpatient care. They stressed how important it was to keep MHS running in such an emergency situation and to evaluate closely which security measures were helpful for their users. Professionals highlighted how the pandemic caused rapid improvements. For instance, digital and telemental health services were extended and their funding secured by health insurances. Interview participants reported on several learning effects evoked by the pandemic: the importance of being flexible as a professional, of managers communicating quickly and clearly, the potential of home treatment, and the need to keep service websites up-to-date. Furthermore, professionals described that due to the pandemic there was an increased awareness for uncontrollable life events in the general population and thus, an improved comprehension for people suffering from mental illnesses. Finally, participants emphasised the capacity to deal with crisis situations and resistance of service users as a positive experience during the pandemic.

### Wishes for future emergency situations

In line with the finding that digital infrastructures were insufficient, the quantitative data showed that participants felt improvements in digitalisation for users (76%) and services (70%) were needed. Furthermore, 64% wished for more staff in MHC, and 70% for ongoing vocational training. 54% indicated the need for more stable funding of their services, and for more clarity in funding of emergency equipment.

In the interviews, participants highlighted the need to take service users and relatives’ experiences into consideration when reacting to emergency situations and to focus measures on vulnerable groups, such as children. They wished for consistent and clearer communication from the policy level and that mental health would be taken into account when developing pandemic guidelines. Professionals recommended guaranteeing the financial support of MHC, improving and maintaining low-threshold access to services, and wished for more therapeutic facilities. Professionals felt that reflection was needed on how to maintain MHS, and in particular therapeutic groups, functioning in such a crisis situation. Finally, they highlighted the necessity to invest in outpatient care as the way forward, as outpatient care proved more flexible in exceptional circumstances than inpatient settings.

### Four different phases of change during the pandemic

In the interviews, professionals stressed that they had experienced different phases of change during the pandemic. The first phase which encompassed the first “lockdown” in Germany from March to May 2020 was described as a phase of shock, great uncertainty, and of rigid measures: “The first phase was quite shocking and quite frightening for us, namely because […] there was the general regulation, the regulation for the residential facilities, the regulation for the workshops, the regulation for care and the like. I always had to read three or four regulations every time something new was published on Friday afternoon. That went into effect on Monday, of course.” [210505] The second phase was perceived as the summer of 2020 (May until November 2020), during which restrictive measures were reduced, services successively opened up again and the feeling of returning to “normality” emerged. The third phase referred to the increasing restrictions and “lockdown” in November 2020 until March 2021, during which professionals saw a “COVID-19 fatigue” happening with high levels of stress, fear and strain. The fourth phase from April 2021 onwards started with the beginning of the vaccination and the re-uptake of services leading to feelings of relief.

## Discussion

The present mixed-methods investigation describes changes faced by MHS due to the COVID-19-pandemic and associated restrictive measures. As has been reported for different locations around the globe [1–5, 34] MHS in the German city of Leipzig were hit unpreparedly by the pandemic and strongly affected. Some institutions closed completely for some time, whereas others experienced discontinuation in their services by having to reduce their offers, particularly concerning the work with therapeutic groups [35]. Infection-control measures were implemented [36], but were staff-intensive and the covering of their additional costs often remained unclear. COVID-19 regulations were experienced as confusing and equivocal by MHC professionals [4].

Noteworthy are the adjustments that have been made in outpatient and community services in view of the present pandemic. Our results indicated that outpatient services, even though facing considerable administrative problems, unclear regulations and rapidly changing restrictive measures, managed to adapt quickly to continue providing adequate support for people with severe mental illness. In other countries, such as China or the UK, services with an extended community mental health infrastructure, particularly those providing a combination of psychosocial and clinical support, have also been reported to respond in a more flexible and adaptive manner to COVID-19 [37, 38]. Considering the risk of clusters of infections in institutions, the expansion of home treatment during the acute phase of the pandemic has been recommended and implemented in many regions [5, 36].

In line with much of the international literature [4, 6, 39–41], the most prominent structural change concerned the digitalisation strategies employed rapidly by MHS. This was generally seen as a positive change. While deficiencies in the digital infrastructure were reported for both, services and users, the present study also highlighted how the COVID-19-pandemic caused an instant upgrading of digital infrastructures and improvement of digital competencies among staff. Clearly, more research is needed investigating the effects of digitalisation in MHC delivery, for instance concerning the question of changes to the quality of the relational experience in digital therapy [34], or issues of privacy, reimbursement and prescription of medication [36].

Our study identified various phases of change during the pandemic. While the first strong restrictions from 22nd March 2020 to 4th May 2020 created uncertainty and feelings of disorganisation and confusion, the second “lockdown” from November 2020 resulted in somewhat more of a feeling of “fatigue” of the pandemic topic and situation. The phases perceived by participants can be seen in direct relation to the changes in policies in Germany [11, 42]. During the different phases, users and professionals were perceived to face diverse challenges and strains.

Users had to deal with the pandemic itself as a topic creating anxieties and worries, with closed or changed services and their higher threshold access, as well as cope with reduction in social contacts. This development paralleled an increasing impairment of service accessibility with growing waitlists and newly introduced pre-registration, which has also been reported by others [4, 5, 34, 36, 39, 41]. Our results suggest that MHS users with chronic illnesses, single parents and elderly users were particularly hampered by the pandemic in their use of MHS. Others have also identified these user groups, as well as people living alone or in conflictual families and healthcare professionals as highly vulnerable to the consequences of the pandemic [4, 41, 43]. The reported impacts of the COVID-19-pandemic on people with severe mental illness range from loneliness, over elevated symptoms of depression and anxiety, to insomnia and post-traumatic stress disorder [5, 44]. As participants in our study, the international literature expects a surge in service users due to the pandemic in the near future [10, 36]. In particular, social participation and inclusion of people with severe mental illness is likely to become even more difficult [5].

Professionals also reported decreases in their own well-being, increased burden, and changes in team dynamics with a worsening in the team atmosphere. Healthcare professionals have been shown to be particularly affected in their well-being during the COVID-19-pandemic [1, 43]. Team activities decreased due to the pandemic, or were transferred to the digital sphere, but working remotely can be a challenge for maintaining team cohesion [45]. Yet, research has shown the importance of teamwork, particularly in high strain settings [46]. Similarly, the importance of having a network of interdisciplinary professionals has been stressed for work in complex and difficult contexts of MHC [47] and also been found in our study. Fortunately, in the city of Leipzig, the MHC sector has been reported to already rely on such a network. Meanwhile, efforts are needed to consolidate this network and improve the interconnectedness with and in rural settings where services are sparse [48]. Furthermore, the present study showed that vocational training was put on hold in most services. However, continuous vocational training, even in exceptional circumstances as a pandemic, seems necessary to increase professionals’ feelings of being prepared and to better service quality.

### Limitations and future research

Given the unprecedented situation, velocity and impact of changes due to the COVID-19-pandemic, we aimed to gain a broad overview of experiences, but much detail has likely been missed. The survey was an ad hoc developed and not yet validated instrument. Our sample may over-represent professionals who are particularly engaged in working towards change and who have many concerns about the current situation. We managed to include a range of professions and work settings, but did not recruit professionals working in private practise. Further efforts to engage and form partnerships are likely to be needed here too. Finally, Leipzig also appears to have many functional MHS and networks in place and rural areas may face very distinctive challenges. Future research could benefit from comparisons of different types of regions (e.g. urban vs. rural) to better understand the effects of the pandemic on MHS. Service user should also be taken into consideration. As further data become available, future research could measure impacts on the mental health system more systematically and making international comparisons.

### Practical implications

In terms of implications, the present study raises attention about the importance of clear communication in policies and infection-control measures, whereby people’s mental health as well as MHS should play an explicit role. Budgets for crisis situations should be established that can cover additional costs for implemented measure and, if needed, extra staff. This, as well as maintaining vocational training and providing adequate supervision for staff is likely to support professionals’ well-being.

The COVID-19-pandemic has shown that community and outpatient services are particularly adaptive to crisis situations [37, 38]. Efforts should be made to foster this specific type of MHC which has also been reported to hold higher responsiveness than inpatient care [49]. Furthermore, this study has shed light on the importance of well-established networks among services. In the future, it will become essential to sustain such networks by creating positions for professional network coordinators and by providing up-to-date overviews of available services for professionals and service users.
